# Mineral nutrients in plants under changing environments: A road to future food and nutrition security

**DOI:** 10.1002/tpg2.20362

**Published:** 2023-07-21

**Authors:** M. Iqbal R. Khan, Faroza Nazir, Chirag Maheshwari, Priyanka Chopra, Himanshu Chhillar, Nese Sreenivasulu

**Affiliations:** ^1^ Department of Botany Jamia Hamdard New Delhi India; ^2^ Division of Biochemistry ICAR—Indian Agricultural Research Institute New Delhi India; ^3^ Consumer‐Driven Grain Quality and Nutrition Center, Rice Breeding and Innovation Platform International Rice Research Institute Los Banos Philippines

## Abstract

Plant nutrition is an important aspect that contributes significantly to sustainable agriculture, whereas minerals enrichment in edible source implies global human health; hence, both strategies need to be bridged to ensure “One Health” strategies. Abiotic stress‐induced nutritional imbalance impairs plant growth. In this context, we discuss the molecular mechanisms related to the readjustment of nutrient pools for sustained plant growth under harsh conditions, and channeling the minerals to edible source (seeds) to address future nutritional security. This review particularly highlights interventions on (i) the physiological and molecular responses of mineral nutrients in crop plants under stressful environments; (ii) the deployment of breeding and biotechnological strategies for the optimization of nutrient acquisition, their transport, and distribution in plants under changing environments. Furthermore, the present review also infers the recent advancements in breeding and biotechnology‐based biofortification approaches for nutrient enhancement in crop plants to optimize yield and grain mineral concentrations under control and stress‐prone environments to address food and nutritional security.

## Core Ideas

- Mineral nutrients mediate defense responses in plants under stressful environments.
- Biofortification enriches mineral nutrient dynamics and crop productivity under changing environments.
- Optimization of nutrient acquisition through genetic engineering addresses food and nutritional security.

**Abbreviations**
AFLP: amplified fragment length polymorphism
APX: ascorbate peroxidase
CaM: calmodulin
CaMKs: CaM kinase
CAT: catalase
CBLs: calcineurin B‐like protein
CCaMK: Ca^2+^/CaM‐dependent protein kinases
CDPKs: CaM‐independent protein kinases
CIPKs: CBL‐interacting protein kinases
DHAR: dehydroascorbate reductase
GR: glutathione reductase
GST: glutathione *S*‐transferase
GWAS: genome wide association studies
H_2_PO_4_
: dihydrogen phosphate
IPK1: inositol‐1,3,4,5,6‐pentakisphosphate 2‐kinase
ITPK: inositol triphosphate kinases
MAB: marker‐assisted breeding
MAS: marker‐assisted selection
MDA: malondialdehyde
MDHAR: monodehydroascorbate reductase
NAS: nicotinamide synthase
NH_4_^+^
: ammonium ion
NO: nitric oxide
NO_3_^−^
: nitrate
PEPRKs: phosphoenolpyruvate carboxylase kinase–related kinases
POD: peroxidases
QTLs: quantitative trait loci
RAPD: randomly amplified polymorphic DNA
RFLP: restriction fragment length polymorphism
ROS: reactive oxygen species
SNP: single nucleotide polymorphism

## 1 INTRODUCTION

Over the last century, the occurrence of famine and rapid increase in the global population has created food insecurity. To overcome these challenges, high yielding varieties in cereals were introduced in combination with the application of higher nitrogen/nutrient applications to soils results in higher productivity, and as a result green revolution was achieved. However, the increase in the global population has led to intensive pressure on natural resources leading to severe climatic fluctuations that affect the crop production. Changing climate has a consequence to deteriorate plant nutritional quality and, thus, has resulted in the global issue of malnutrition among the population (FAO, 2017).

Plants need six major macro minerals, such as nitrogen (N), phosphorous (P), potassium (K), magnesium (Mg), sulfur (S), and calcium (Ca), and micronutrient minerals covering iron (Fe), zinc (Zn), manganese (Mn), copper (Cu), boron (B), and molybdenum (Mo) that are essential for driving plant growth and development. Nutrients act as an integral component of the plant system that plays a significant role in coordinating several physiological and developmental aspects in plants such as seed germination, root and shoot growth, photosynthesis, respiration, as well as flower development (Kirkby et al., 2023; Saleem et al., 2023). Mineral nutrients help plants yield better by supporting physiological processes, such as photosynthesis, respiration, and transpiration. The uptake of most mineral nutrients (Fe, Zn, Mn, and Mo) decreased under abiotic stresses (D'Oria et al., 2022; Giri et al., 2017).

To ensure nutrition security, the production of crop plants with high nutritive value is required. Biotechnologists and breeders have been consistently working on deciphering the molecular and physiological determinants of plant nutrition under optimal as well under stress‐prone conditions. Several stress‐responsive genes (including mineral transporters, aquaporins, and vacuole transporters) that regulate nutrient uptake, accumulation, and their metabolism in plants have been identified and manipulated by genetic engineering approaches to produce transgenics with improved nutritive values (Bao et al., 2015, 2009; Zhang, Hong, et al., 2020). Biofortification using molecular plant breeding approaches has also been undertaken to address the issues of malnutrition and ensure nutrition and food security (Calayugan et al., 2020). To this end, efforts are made for producing crops with enhanced nutritional values by identifying quantitative trait loci (QTLs) using the traditional QTL mapping approach (Calayugan et al., 2020; Jeong et al., 2020), or by employing the genome wide association studies (GWAS) for identifying high value genes for enriched minerals and vitamins in grains (Lopes et al., 2015; Pariasca‐Tanaka et al., 2020). Crops, such as pearl millet (*Cenchrus americanus*) with enriched Fe, maize (*Zea mays*), and rice (*Oryza sativa*) with increased vitamin A, wheat (*Triticum aestivum*), and rice varieties with elevated grain Zn, have been developed through biofortification approaches using biotechnological and breeding approaches to address nutritional security (Bouis & Saltzman, 2017).

**Core Ideas**

- Mineral nutrients mediate defense responses in plants under stressful environments.
- Biofortification enriches mineral nutrient dynamics and crop productivity under changing environments.
- Optimization of nutrient acquisition through genetic engineering addresses food and nutritional security.

Together with the concerted efforts of traditional breeding and marker‐assisted selections (MAS) more than 400 varieties of conventionally bred biofortified, 12 crops have released in 40 countries through the concerted efforts of HarvestPlus, several CGIAR institutions, and participating collaborative national partners (https://www.harvestplus.org/crop‐development/). These biofortification approaches are bred with a targeted increment of Fe content in beans (44 ppm), Zn content in pearl millet (30 ppm), rice (12 ppm), maize (12 ppm), and wheat (18 ppm) (Van Der Straeten et al., 2020). To be successful in biofortification approaches through breeding, enough intraspecies genetic variation needs to be identified. To overcome these limitations, biotechnology‐based biofortification approaches such as single‐gene engineering methods or a modular design of metabolic pathway engineering to overexpress the dominant effect genes or to undertake the genome editing approaches to target the recessive genes to fine‐tune or lower the expression and activity of the introduced enzymes are undertaken. These interventions are expected to provide more efficient solutions to enrich micronutrients in the edible parts of the crops; however, the policy interventions and regulatory compliances are required to explore the benefits of human health.

In this review article, we highlighted efforts (i) to bring out the importance of mineral nutrients in plants under environmental fluctuations to efficiently optimize nutritional dynamics and plants performance, and (ii) recent advancements in plant biotechnology and molecular breeding programs to assist biofortification with high productivity under ambient and unfavorable environmental conditions. To address future food and nutritional security issues in the changing climatic scenario, it is critical to pyramid the strategies between the two approaches to maintain plant health by increasing the mineral uptake under abiotic stress‐prone environments and to identify mechanisms of source–sink alteration to enrich multiple minerals in the edible grain to promote sustained human health.

## 2 IMPORTANCE OF MINERAL NUTRIENTS IN PLANTS UNDER STRESSFUL ENVIRONMENTS

Essential mineral elements, such as N, carbon (C), and P, are required in relatively higher amounts as they are the constituents for building fundamental biomolecules, such as amino acids (Khan et al., 2019), nucleotides, and carbohydrates, and play an important role in achieving maximum grain yield. Essential mineral nutrients such as K and Mg play a crucial role in important plant physiological processes such as photosynthesis and translocation of photo‐assimilates (Xie et al., 2021). However, K functions, as a major osmolyte, regulate guard cell activities and stomatal conductance (Jákli et al., 2017). Mg is the major requirement for the absorption of light, the synthesis of chlorophyll *a* and *b*, and the activation and abundance of Rubisco and Rubisco activase (Peng et al., 2015). In plants, mineral elements also play an essential role as cell structure constituents, in turgor‐related processes, plant reproductions, and energy‐transfer reactions (Pandey, 2018). Similarly, essential micronutrients, such as Fe and Zn, are also essential components of various plant physiological processes, such as photosynthesis, reactive oxygen species (ROS) scavenging, signaling, gene expression, and growth hormone production. Thus, maintaining adequate levels of Fe and Zn in plants is very important for ensuring normal plant growth and adequate crop yield. Controlling the expression of master regulators that regulate the plant responses during low nutrient conditions can enhance nutrient uptake and acquisition. Boron (B) is another essential trace element that is involved in maintaining plant cell wall integrity, ion fluxes across the membrane, carbohydrate metabolism and growth of the plant (Shireen et al., 2018). It also affects uptake, translocation and availability of other mineral nutrients such as K, P, Zn, and Cu in different parts of the plant (Shireen et al., 2018).

**FIGURE 1:**
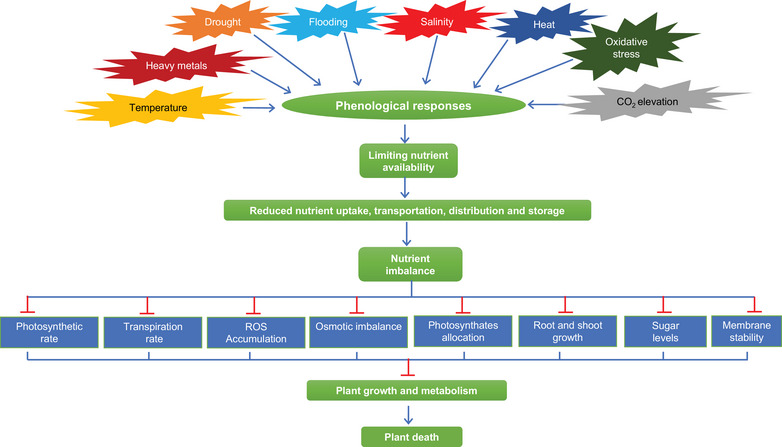
Phenological responses of plants to nutrients during unfavorable environmental conditions. During abiotic stress conditions, various morphological, physiological, biochemical and molecular alterations occur in plants. The changes in nutritional dynamics and disbalance in nutrient homeostasis is the major factor limiting plant growth, development, and yield during such conditions. Abiotic stress conditions limit nutrient availability thereby reducing nutrient acquisition, distribution, and storage that ultimately leads to deficiency of macro and micronutrients in plants. Such changes in nutritional dynamics results in deterioration of plants growth and metabolism in terms of physiological and morphological traits such as photosynthetic and transpiration rate, oxidative damage, membrane instability, and so on that ultimately leads to plant death.

When plants take up minerals from stress‐prone soils help to cope with its plant growth and thereby these mineral elements act as stress alleviators that mitigate abiotic stress‐induced adversities in plants (Huang et al., 2019; Lotfi et al., 2022; Zhang et al., 2017) and ensure their better growth and survival during harsh conditions (Table 1; Figure 1). In the following sections, we discussed the importance of macro‐ and micronutrient minerals that are essential to promote plant health under changing climates.

**TABLE 1:** Representative studies on the tapped potential of mineral nutrients in elucidating multiple responses to develop abiotic stress resilience in crop plants.

Plant species	Mineral nutrients (source, concentration, and their mode of application)	Abiotic stress and its condition	Responses	References
*Brassica juncea* (mustard)	KNO_3_ (N; 10 mM) through nutrient solution	Salinity stress (NaCl; 100 mM)	Improved photosynthetic rate and osmotic balance by regulating proline and ethylene production	Iqbal et al. (2015)
*Triticum aestivum* (wheat)	Urea [CO(NH_2_)_2_, sodium nitrate (NaNO_3_ (NO_3_^−^)), or ammonium chloride (NH_4_Cl (NH_4_^+^)); 750 kg h^−1^] through foliage	Drought stress water potential at the 15‐cm soil layer (−60 ± 5 kPa)	Accelerated the grain filling rate by regulated endogenous phytohormones (ABA, *z*‐riboside (ZR), and ethylene), the antioxidant enzyme system (SOD, POD, and CAT) in flag leaves, and gene expression (*AGPP‐L*, *GBSSI*, *SSSI*, *SSSII*, *SBEIIa*, and *SBEIIb*) involved in remobilization of carbohydrates and starch synthesis in wheat grains and thus alleviated the inhibitory effects of drought stress	Lv et al. (2021)
*Brassica oleracea* (cauliflower)	(KNO_3_/NH_4_)_2_SO_4_ (NO_3_^−^/NH_4_^+^; 50:50) through nutrient solution	Heat stress (43/30°C)	Regulate antioxidant levels, optimize photosynthetic rate, and its related variables and uphold nutrient homeostasis	Collado‐González et al. (2021)
Wheat	Urea (N; 1.48 g pot^−1^) mixed with soil	Heat (36/26°C) and drought stress (75%–85% SWHC)	Influences photosynthesis, *N*‐metabolism, yield, and water and nitrogen use efficiency	Ru et al. (2022)
*Brassica napus* (rapeseed)	N (30, 60, and 90 kg ha^−1^) mixed with soil	Waterlogging stress (imposed for 6 and 9 days)	Enhanced the accumulation of dry matter and N in which might be attributed to the strong antioxidant defense system, maintenance of photosynthetic pigments, and the nutrient balance	Men et al. (2020)
*Glycine max* (soybean)	NH_4_H_2_PO_4_ (P; 60 and 120 mg kg^−1^) mixed with soil	Drought stress (SRWC; 30%)	Reinforcing root growth and enduring daily water use during flowering and podding, which had a strong outcome on flower number filled‐pod number, and grain yield	He et al. (2019)
Mustard	(NH_4_)_2_HPO_4_ (P; 30 mg kg^−1^) mixed with soil	Na_2_HAsO_4_ (As; 8, 16, or 24 mg kg^−1^)	Alleviated As toxicity by influencing NO biosynthesis, enhancing proline metabolism, ROS scavenging systems such as ascorbate–glutathione and glyoxalase system, and improved plant growth	Khan et al. (2021)
*Oryza sativa* (rice)	Phosphate (3 mM) through nutrient solution	CdCl_3_ (Cd; 8.896 and 100 μM)	Reduced Cd accumulation and enhance Cd resistance in rice by affecting the expression of Cd transporter (*OsHMA2*, *OsIRT1*, and *OsABCC1*) and signaling molecules (*OsIAA17*, *OsACO*, and *OsNR2*) genes	Chen et al. (2022)
Rice	Ca (H_2_PO_4_)_2_ (P; 2 g) mixed with soil	High temperature (35 ± 2°C)	Highest grain production and better grain quality, which might be due to enhanced photosynthesis, WUE, and grain size, which compensated the adversities of high temperature stress	Fahad et al. (2016)
*Solanum tuberosum* (potato)	Potassium phosphite (1%) through foliage	Heat stress (37/29°C)	Ameliorates the adverse effects of heat stress and enhances thermotolerance by repressed heat stress perception, which triggered ROP for signal transduction, activation of genes, which attributed to scrounge excess ROS, acquisition of stress proteins, which implied changes in metabolic processes, and augment biosynthesis of osmolytes, and defense metabolites, thereby sustaining osmotic adjustment	Xi et al. (2020)
Wheat	Superphosphate (P; 5 g, base application 2.5 g + top application 2.5 g at jointing stage)	Cold stress (4°C, chilling and −4°C, freezing)	Strengthening soil nutrition relationship, enhancing photosynthetic capacity, slowing down flag leaf senescence, ensuring source–sink balance, and inevitably curtailing yield losses	Xu et al. (2022)
Soybean	K_2_SO_4_ (K; 9 mM) through nutrient solution	Salinity stress (NaCl; 9 mM)	Restored plant growth by augmenting antioxidant defense, photosynthetic efficiency, carbohydrates accumulation, yield, and seed quality traits	Parveen et al. (2021)
*Brassica campestris* (pak choi)	ZnSO_4_ (Zn; 25 μM) through foliage	Salinity stress (NaCl; 80 mM)	Lowered oxidative damage and elevated the level of glucosinolates, which could act as important signals to improve water uptake and transport	Fatemi et al. (2020)
Wheat	ZnSO_4_ (0.1% and 0.2%) through foliage	Drought (60% FC)	Increased the chlorophyll contents and WUE and eventually increased productivity and grain nutrient content	Anwar et al. (2021)
*Zea mays* (maize)	Zn (6 μM)	Drought	Improved root structural integrity as a result of enhancements in the transcription of *ZmPIP1;1*, *ZmPIP1;2*, and *ZmPIP2;2* (aquaporin gene expression) genes	Zhang, Yan, et al. (2021)
Wheat	ZnSO_4_ (Zn; 8 mM) through foliage	CdCl_2_ (Cd; 0.5 mM)	Amplify antioxidant system components and osmolytes, as well as greatly reduce membrane lipid peroxidation	Perveen et al. (2020)
Wheat	ZnSO_4_ (Zn; 0.2–2.0 mg^−1^) through nutrient solution	Heat stress (40/20°C)	Enhanced biomass production, maximum yield of PSII, chlorophyll fluorescence, and kernel growth	Peck and McDonald (2010)
Wheat	ZnSO_4_ (Zn; 15 mg kg^−1^) mixed with soil	Heat stress (20°C for 2 days)	Improved yield and quality traits	Tao et al. (2018)
Rice	ZnSO_4_ (Zn; 0.31 μM) through nutrient solution	Cold stress (12 and 20°C)	Promoted rice tiller recovery by increasing N and Zn accumulation and maintaining hormones balance	Liu et al. (2022)
Rice	ZnSO_4_ (Zn; 10 mg kg^−1^) mixed with soil	Flooding stress (imposed for 15 days)	Promoted growth and yield traits after complete submergence	Singh and Singh (2020)
Maize	K_2_SO_4_ (H_2_S; 40 mM) through foliage	Salinity stress (NaCl; 25, 75 mM)	Increased photosynthetic attributes and the concentration of biomolecules	Riffat et al. (2020)
*B. napus* (canole)	SO_4_^2−^ (S; 1 mM) through nutrient solution	Chromium stress (CrVI; 100 μM)	Impacted Cr accumulation and enhanced tolerance caused by a positive effect on defense systems and expression level of metallothionein gene (*BnMP1*)	Terzi and Yildız (2015)
Rice	SO_4_^2−^ (S; 0.5, 3.5, and 5.5 mM) through nutrient solution	NaAsO_2_ (AsIII; 25 μM)	Restricted As accumulation in a shoot by boosting thiol metabolism and glycolysis toward amino acid accumulation	Dixit et al. (2015)
Wheat	Na_2_SO_4_ (S; 10 mM) through nutrient solution	MnCl_2_ (Mn; 3 mM)	Potentiating antioxidant defense, as well as the remobilization and allocation of Mn from roots to shoots, excess Mn sequestering in vacuoles in the form of PCs, and elevated levels of GSH	Sheng et al. (2016)
*Solanum lycopersicum* (tomato)	S (6 ppm) through foliage	Heat stress (45 ± 2°C)	Alleviate heat stress in tomato by increasing the proline production and cellular redox homeostasis under heat stress	Ali et al. (2021)
Soybean	CaCl_2_ (Ca; 6 mM) through nutrient solution	Salinity stress (NaCl; 50 mM)	Increased salt stress tolerance of germinating soybeans by empowering signal transduction, energy pathway and transportation, facilitating protein biosynthesis, impeding proteolysis, restricting protein processing in endoplasmic reticulum, stimulating antioxidant enzymes and amplifying their activities, piling up secondary metabolites and osmolytes	Yin et al. (2015)
Rice	CaCl_2_ (Ca; 100 μM) through nutrient solution	K_2_Cr_2_O_7_ (100 μM)	Induced PCs accumulation and regulation of antioxidant defense	Mukta et al. (2019)
Wheat	Ca (10 mM) through foliage	Heat stress (42°C, 2 h)	Improved tolerance due to an enhanced transcript level of SAGs; *CDPK*, *HSFA4a*, *HSP17*, *SOD* and *APX* and triggers the activity of kinases and peroxidases	Goswami et al. (2015)
Maize	CaCl_2_ (Ca; 80 mmol L^−1^) seed priming	Cold stress (<10°C)	Safeguard the structure and functionality of the membrane and photosystems, strengthen antioxidant enzyme activity, and increase osmotic regulatory substances	Zhang, Liu et al. (2020)
Rapeseed	SiO_2_ (Si; 1 mM) through soil	Drought stress (PEG; 10% and 20%)	Protected photosynthetic pigments, reduced ROS accumulation via enhanced activities of glyoxalase enzymes, increased relative water content, proline content, and activities of antioxidant enzymes	Hasanuzzaman et al. (2018)
Wheat	Sodium silicate (Si; 204 mg kg^−1^) mixed with soil	Drought stress (21.9% FC)	Enhanced the contents of AsC and GSH, and the total phenolic and total flavonoid contents along with increased AsA–GSH cycle genes (*TaMDHAR*, *TaDHAR*, *TaGS*, and *TaGR*), and flavonoid biosynthesis pathway genes (*TaPAL*, *TaCHS*, *TaF3H*, *TaDFR*, and *TaANS*)	Ma et al. (2016)
Rice	Nanoscale silica hydrosol (SiO_2_; 5 mmol L^−1^) through foliage	CdCl_2_ Cd (1.0 μmol L^−1^)	Boosting the expression of genes related with essential nutrient transporters, carbohydrate and secondary metabolite biosynthesis, and cytochrome oxidase activity in Cd‐exposed flag leaves and spikelets, which could assist in alleviating oxidative stress and promote plant growth	Sun et al. (2022)
Tomato	Na_2_SiO_3_ (Si; 1 mM) through foliage	Heat stress 43 ± 0.5 °C	Evoked stress tolerance by potentiating heat transcription factors (Hsfs) such as *SlHsfA1a‐b*, *SlHsfA2‐A3*, and *SlHsfA7*, as well as endogenous phytohormones (SA and ABA) and related mRNA gene expression patterns	Khan et al. (2020)
*Hordeum vulgare* (barley)	Na_2_SiO_3_ (Si; 56 mg L^−1^) through nutrient solution	Cold stress (+ 25 to + 5°C, −5°C)	Manipulating the activity of antioxidative enzymes and the concentrations of soluble carbohydrates and proteins in the leaf apoplast	Joudmand and Hajiboland (2019)
Rice	K_2_SiO_3_ (Si; 2 mm kg^−1^) mixed with soil	Flooding stress (imposed for 7 days)	Boosting Si uptake and acquisition, improving root morphological traits and chloroplast ultrastructure, and amplifying antioxidant defense	Pan et al. (2021)

Abbreviations: ABA, abscisic acid; AlCl_3_, aluminum chloride; APX, ascorbate peroxidase; As, arsenic; AsC, ascorbate; BR, brassinosteroid; [Ca (H_2_PO_4_)_2_, triple superphosphate; C_7_H_5_KO_3_, potassium salicylate; Ca, calcium; CaCl_2_, calcium chloride; CaNO_3_, calcium nitrate; Cd, cadmium; CdCl_2_, cadmium chloride; CDPKs, CaM independent protein kinases; Cr, chromium; Cys, cysteine; ECW, electrical conductivity of water; FC, field capacity; Gly, glycine; GOGAT, glutamine oxoglutarate aminotransferase; GSH, glutathione; H_2_S, hydrogen sulfate; HSF, heat shock factor, HSP, heat shock proteins; K_2_Cr_2_O_7,_ potassium dichromate; K_2_SiO_3_, potassium silicate; K_2_SO_4_, potassium sulphate; KH_2_PO_4_, potassium monophosphate; KNO_3_, potassium nitrate; Mn, manganese; MnCl_2,_ manganese chloride; N, nitrogen; Na_2_HAsO_4_, sodium arsenate; Na_2_SiO_3_, sodium silicate; NaCl, sodium chloride; NaHS, sodium hydrosulfide; NH_4_^+^, ammonium; NH_4_HCO_3_, ammonium bicarbonate; (NH_4_)_2_HPO_4_, ammonium dihydrogen phosphate; NH_4_NO_3_, ammonium nitrate; NO_3_^−^, nitrate; NR, nitrate reductase; PC, phytochelatins; PEG, polyethylene glycol; POD, peroxidase; ROP, rho of plants; ROS, reactive oxygen species; SA, salicylic acid; SAG, stress‐associated genes; SF3BI, splicing factor 3 B subunit 1‐like; Si, silicon; SiO_2_, silicon dioxide; SO_4_^2−^, sulfate ion; SOD, superoxide dismutase; SOS, salt overly sensitive; SWHC, soil water holding capacity; TCA, tricarboxylic acid cycle; WUE, water use efficiency; ZFP, zinc finger protein; Zn, zinc; ZnSO_4_, zinc sulfate.

### 2.1 Nitrogen

The availability of N (10 mM) successively alleviates salinity‐induced adversities in mustard (*Brassica juncea*) and improves plant photosynthetic rate and osmotic balance by regulating proline and ethylene production (Iqbal et al., 2015). Recently, it is has been shown that various forms of foliar N [NO_3_^−^, ammonium (NH_4_^+^), and urea (CO(NH_2_)_2_)] at the rate of 750 kg ha^−1^ increased wheat grain filling rate by modulating endogenous phytohormones, the antioxidant enzyme system in flag leaves, and gene expression implicated in remobilizing carbohydrates and starch synthesis in grains and thus mitigated the deleterious effects of drought stress (Lv et al., 2021). Exogenous application of different ratios of NO_3_^−^/NH_4_^+^ (50:50) has been shown to regulate antioxidant levels, optimize photosynthetic rate, and its related variables and maintain nutrient homeostasis in heat exposed cauliflower (*Brassica oleracea*) plants (Collado‐González et al., 2021). Furthermore, high‐N supply (8 mM) alleviated cold stress‐dependent growth inhibition in maize seedlings by increasing photosynthesis, N assimilation, carbohydrate partitioning, and improving redox homeostasis (Soualiou et al., 2023). After waterlogging, the application of N fertilizer (30, 60, and 90 kg ha^−1^) enhanced the accumulation of dry matter and N content in rapeseed (*Brassica napus*), which may have contributed to the strong antioxidant defense system, restoration of photosynthetic pigments, and nutrient homeostasis (Men et al., 2020), resulting in improved rapeseed growth recovery. Thus, sustainable N management and improved nitrogen use‐efficiency (NUE) are critical to resolving the global ecological crisis while ensuring adequate food supply.

### 2.2 Phosphorous

**FIGURE 2:**
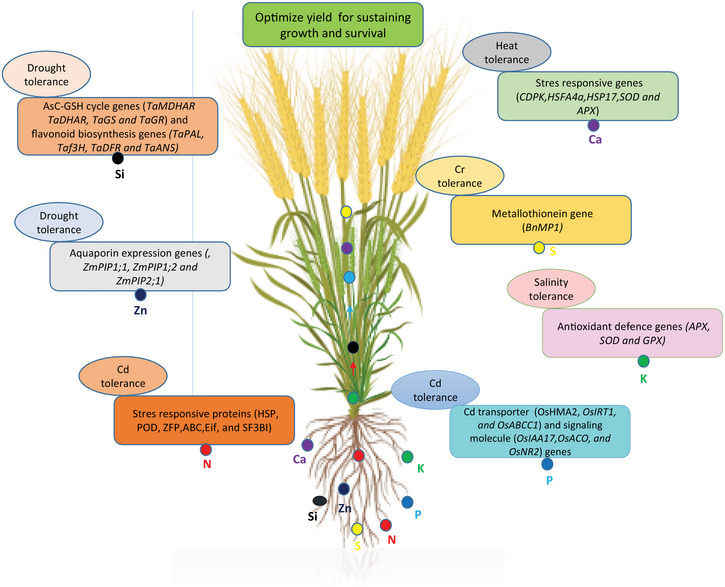
Exogenously applied mineral nutrients triggers several metabolic and molecular adaptations in plants to mitigate the stress‐induced adversities and readjust nutrient pools for sustaining plant growth and survival under such harsh conditions, thereby addressing food and nutritional security. APX, ascorbate peroxidase; AsA, ascorbate; Cd, cadmium; Cr, chromium; e1F, eukaryotic translation initiation factor; GSH, glutathione; HSF, heat shock factor; HSP, heat shock protein; POD, peroxidase; SF3BI, splicing factor 3 B subunit 1‐like; SOD, superoxide dismutase, ZFP, zinc finger protein. Strategies for crop improvement to enhance nutritional levels for sustainable agriculture. Several strategies have emerged for crop improvement to enhance nutritional levels in plants to cope with nutritional deficiency during abiotic stress conditions for sustainable agriculture. Biotechnological interventions backed up by biofortification of crops and other agronomic strategies have been employed for producing nutritionally rich crops with enhanced tolerance to abiotic stresses. Biotechnological approaches involve identification and manipulation of genes, proteins and metabolites associated with plant nutrition, whereas approaches such as quantitative trait locus (QTL) mapping and genome wide association studies (GWAS) have also been employed for biofortification of food crops. Other agronomic traits include the utilization of several nutrient rich fertilizers, manures, foliar application of nutrients and/or different system of agriculture such as intercropping to prevent nutrient deficiency.

Adequate P nutrition also plays a significant role in plant responses and resilience to abiotic stresses. Under drought stress, P (60 and 120 mg kg^−1^) fertilization was found to be effective in promoting root growth and sustaining daily water use during flowering and podding, which had a significant effect on flower number filled‐pod number, and grain yield in soybean during prolonged drought (He et al., 2019). Interestingly, supplementation of P (30 mg P kg^−1^) reduced arsenic (As) toxicity in the mustard plant by influencing NO (nitric oxide) biosynthesis, enhancing proline metabolism, ROS scavenging systems such as ascorbate–glutathione (AsA–GSH) and glyoxalase system, and improving plant growth (Khan et al., 2021). P (3 mM) treatment has been shown to reduce cadmium (Cd) accumulation and improve Cd resistance in rice by downregulating the transcription of Cd transporter (*OsHMA2*, *OsIRT1*, and *OsABCC1*) and signaling molecules (*OsIAA17*, *OsACO*, and *OsNR2*) genes (Chen et al., 2022; Figure 2). This study advances our understanding of the intrinsic regulation of P–Cd interactions in crops. Under high temperature stress, supplementation of P (2 g) had a superlative impact on rice, resulting in higher grain production and better grain quality, which could be attributed to increased photosynthesis, water use efficiency (WUE), and grain size, which recompensed the adversities of high‐temperature stress (Fahad et al., 2016). Furthermore, results from physiological data combined with RNA‐Seq showed that the exogenous application of phosphite (1%) ameliorates the deleterious effects of heat stress and enhances thermotolerance of potato (*Solanum tuberosum*) by suppressing heat stress perception, which stimulated rho of plants for signal transduction, activation of genes, which attributed to scavenge excess ROS, trigger of stress proteins to cope metabolic processes and augment biosynthesis of osmolytes and defense metabolites, thereby sustaining osmotic adjustment (Xi et al., 2020). Under low‐temperature stress, the positive effects of optimized P (5 g) in wheat were evidenced in strengthening soil nutrition relationship, enhancing photosynthetic capacity, slowing down flag leaf senescence, ensuring source–sink balance, and inevitably reducing yield losses (Xu et al., 2022).

### 2.3 Potassium

Under salinity stress conditions, K application (6 and 9 mM) revived plant growth by augmenting antioxidant defense, photosynthetic efficiency, carbohydrates accumulation, yield, and seed quality traits in soybean (Parveen et al., 2021; Taha et al., 2020). Similarly, Lotfi et al. (2022) found that K fertilization (150 kg h^−1^) improves photosynthetic activity, yield, and seed quality in wheat in drought‐prone arid and semiarid regions. Shahid et al. (2020) studied the effect of varying K concentrations, ranging from 15 to 60 g L^−1^ on wheat plants under heat stress, of which the best results from flowering to grain filling such as increased spike length, spikelets per spike, 1000‐grain weight, and grain yield were observed at 45 and 60 g L^−1^ in heat‐stressed rice plants. Thus, maintaining a sufficient K supply can be a significant nutritional strategy for crop plant survival under situations of environmental stresses. During K‐deficient stress conditions, the photosynthetic and transpiration rate were decreased in a K‐deficiency‐sensitive variety GD8521, whereas the tolerant variety Tiefeng 40 of soybean (*Glycine max*) exhibited normal photosynthetic rate and optimal growth with no photo‐oxidative damage during K deficiency (Wang et al., 2015); hence exploring varietal responses to K use efficiency (KUE) is required. In the future, it will be necessary to look into the mechanistic basis evoked by K fertilizer to enhance abiotic stress resilience in plants in order to assure effective fertilizer utilization and food security globally.

### 2.4 Zinc

Likewise, Zn application in low doses has enticing capability for recouping plants that have been confined to abiotic stresses. For instance, in pak choi (*Brassica campestris*) plants, Zn application (25 μM) lowered oxidative damage and elevated the level of glucosinolates, which could act as important signals to improve water uptake and transport and, as a result, ameliorate salinity stress (Fatemi et al., 2020). Furthermore, Zn application (250 M) improved salinity resilience in mungbean (*Vigna radiata*) by increasing the efficiency of secondary metabolism enzymes, resulting in an increase in total phenol and flavonoids, which could be effective in nutrient aided management for crop production (Al‐Zahrani et al., 2021). Under drought stress, foliar Zn (0.1% and 0.2%) application to wheat at both the vegetative and reproductive stages elevated chlorophyll content and WUE, resulting in increased productivity and grain nutrient content in wheat (Anwar et al., 2021). Zn (6 M)‐mediated improvements in root structural integrity in drought‐exposed maize seedlings were linked with the transcription of *ZmPIP1;1*, *ZmPIP1;2*, and *ZmPIP2;2* (aquaporin gene expression) genes (Zhang, Yan, et al., 2021; Figure 2). Exogenously applied Zn has been shown to augment growth and photosynthetic traits in several crop plants, including chromium [Cr (100, 500 mg kg^−1^)] exposed rice (Hussain et al., 2018), Cd (0.5 mM)‐exposed wheat (Perveen et al., 2020), and Cu (200 and 500 μM)‐exposed rice (Thounaojam et al., 2014; Table 1). Furthermore, Zn‐supplementation has been revealed to support several plant species differentially, when confronted with high temperatures (Tao et al., 2018; Table 1). For instance, Tao et al. (2018) recently reported that treatment with 15 mg kg^−1^ Zn can ameliorate the deleterious effects of high‐temperature stress on wheat yield and quality, which may be advantageous to growers desiring ways to maximize grain yield while sustaining grain quality in the light of the potential menaces to plant growth posed by climate change. It has also been reported that Zn (0.31 M) application after low‐temperature‐aided rice tiller recovery by boosting N and Zn accumulation and retaining hormone balance (Liu et al., 2022). According to a recent study, Zn (10 mg kg^−1^) significantly increased the yield of Sub1 and non‐Sub1 rice varieties even after 15 days of total submergence (Singh & Singh, 2020). These findings suggest that mineral nutrient application may ameliorate the inhibitory effect of abiotic stress and improve tolerance by exploiting key processes in plants under abiotic stresses.

### 2.5 Sulfur

S is also an essential macronutrient in plants, where in S (40 mM), application ameliorated the effect of salinity by changing organic and inorganic osmolyte contents in maize plants (Riffat et al., 2020). Similarly, priming with 100 μM sulfur nanoparticles (SNPs) improved photosynthetic pigments, N metabolism, antioxidant status, and ionic relations contributing to the enhancement of growth attributes in wheat under salinity (Saad‐Allah & Ragab, 2020). In addition, the optimized doses of various sources of S (K_2_SO_4_ and Na_2_SO_4_; 30 kg ha^−1^ and CuSO_4_ and FeSO_4_; 45 kg ha^−1^) significantly increased the gas exchange characteristics, nutrient uptake, and activity of antioxidant enzymes, which ultimately improved maize yield under water‐deficit conditions (Usmani et al., 2020). Furthermore, S nutrition (1 mM) affected Cr accumulation and enhanced resilience in canola (*B. napus*) by improving defense systems and the expression level of the metallothionein gene (*BnMP1*) (Terzi & Yildız, 2015; Figure 2). Moreover, Zhang et al. (2019) revealed that soil‐applied elemental S^0^ and gypsum (0.15, and 0.30 g S kg^−1^) effectively mitigated the negative effects of Cd stress on rice, which was attributed to restricted Cd translocation from root to shoot and significantly decreased rice grain Cd concentration, thereby increasing biomass and yield traits. Sheng et al. (2016) revealed that a moderate level of S application (10 mM) protected wheat plants against manganese (Mn) toxicity by enhancing antioxidant defense system, and the remobilization and allocation of Mn from roots to shoots, excess Mn sequestering in vacuoles in the form of phytochelatins (PCs), and elevated levels of GSH, where GSH played a significant role. Recently, Ali et al. (2021) reported that 6 ppm S treatment can mitigate heat stress in tomato by enhancing proline production and cellular redox status under heat stress. Similarly, 600 ppm S supplementation improved physiological and yield traits in heat‐exposed canola plants (Waraich et al., 2022). There are still insufficient reports to explicate the molecular mechanism of S metabolism under abiotic stresses; therefore, regulatory networks of S metabolism could be explored, allowing us to answer the question of how S is metabolized under unfavorable environments.

### 2.6 Calcium

Calcium (Ca) also plays an important role in mediating plant growth and development during environmental cues as it acts as an intracellular messenger, maintains structural and functional integrity of cell membrane, mediates stomatal activity, sap flow, functioning of photosystem II, and ROS concentration under unfavorable conditions (Ahmad et al., 2015; Sakhonwasee & Phinkasan, 2017). According to physiological and comparative proteomic analysis, exogenous Ca (6 mM) could increase salt stress tolerance of germinating soybeans by enhancing signal transduction, energy pathway and transportation, facilitating protein biosynthesis, impeding proteolysis, restricting protein processing in endoplasmic reticulum, stimulating antioxidant enzymes and amplifying their activities, increasing secondary metabolites and osmolytes, and other adaptive responses (Yin et al., 2015). Furthermore, Ca (10 and 20 mM) has been shown to improve salinity tolerance in sorghum (*Sorghum bicolor*) by reducing oxidative damage, governing hormone signal transduction, and regulating the expression of genes (*GR3*.*4*, *GADX1*, *IQM4*, *GR2*.*8*, and *AnnD7*) related to the lignin degradation, absorption of NH_4_^+^, energy metabolism, and salt response (Yang et al., 2022). Exogenously applied Ca (10 mM) has been shown to improve heat tolerance in wheat by increasing the transcript levels of stress‐associated genes such as *CDPK*, *HSFA4a*, *HSP17*, *SOD*, and *APX*, as well as activating kinases and peroxidases (Goswami et al., 2015; Figure 2). In maize, exogenous Ca at a concentration of 80 mmol L^−1^ has been shown to protect the structure and functionality of the membrane and photosystems, strengthen antioxidant enzyme activity, and increase osmotic regulatory substances under cold stress (Zhang, Liu, et al., 2020). These studies employed molecular and physiological mechanisms to explain how Ca alleviates the effects of abiotic stress‐induced adversities on plant growth inhibition, which could lead to a better understanding of the functional mechanistic explanation of how Ca alleviates abiotic stress in plants.

### 2.7 Silicon

Quasi‐essential elements such as silicon (Si) are also important in mediating abiotic stress tolerance in plants and maintaining their physiological, cellular, and functional integrity (Hasanuzzaman et al., 2018; Sun et al., 2022). For instance, exogenous application of Si (204 mg kg^−1^) considerably enhanced the contents of AsA and GSH, and the total phenolic and total flavonoid contents along with increased AsA–GSH cycle genes (*TaMDHAR*, *TaDHAR*, *TaGS*, and *TaGR*), and flavonoid biosynthesis pathway genes (*TaPAL*, *TaCHS*, *TaF3H*, *TaDFR*, and *TaANS*) in wheat, indicating that Si plays a critical role in the synchronized transcriptional activation of myriad antioxidant defenses in response to drought stress (Ma et al., 2016; Figure 2). Besides, Si also alleviated the Fe‐deficiency‐induced damages in sorghum plants by ameliorating Fe transportation and utilization, increasing chlorophyll content in the leaf, along with decreasing malondialdehyde (MDA) content (Teixeira et al., 2020). Foliar spray of Si (5 mmol L^−1^) also ameliorated Cd stress‐induced damage in rice by boosting the expression of genes related with essential nutrient transporters, carbohydrate and secondary metabolite biosynthesis, and cytochrome oxidase activity in Cd‐exposed flag leaves and spikelets, which could assist in alleviating oxidative stress and promoting plant growth under Cd (Sun et al., 2022). Another study uncovered that applying Si (1 mM) to tomato seedlings improved the resilience and activity of tomato (*Solanum lycopersicum*) plants under heat stress and evoked stress tolerance by potentiating heat transcription factors (Hsfs) such as *SlHsfA1a‐b*, *SlHsfA2‐A3*, and *SlHsfA7*, as well as endogenous phytohormones (salicylic acid and abscisic acid) and related mRNA gene expression patterns (Khan et al., 2020). Furthermore, Si (65 mg L^−1^) protects barley (*Hordeum vulgare*) plants from cold stress by influencing the activity of antioxidative enzymes and the concentrations of soluble carbohydrates and proteins in the leaf apoplasm (Joudmand & Hajiboland, 2019). The supplementation of Si (2 mm kg^−1^) to rice plants could mitigate the deleterious effect of submergence stress by boosting Si uptake and acquisition, improving root morphological traits and chloroplast ultrastructure, and amplifying antioxidant defense (Pan et al., 2021). Thus, Si can be considered a highly beneficial element that significantly ameliorate stress‐induced damage, thereby facilitating plant survival. More research is required to comprehend the molecular mechanisms of Si‐alleviated abiotic stresses.

### 2.8 Cross‐talk between mineral nutrients in crop plants

There has been little research into how plants integrate multiple nutrient signals into developmental programs, as well as the molecular processes underlying these complex cross‐talks. For instance, in the roots of barley and Arabidopsis (*Arabidopsis thaliana*), there is competition for NH_4_^+^ and K uptake. Plant K^+^ transporters and channels were shown to transport NH_4_^+^. As a result, NH_4_^+^ uptake via K^+^ transporters may contribute to NH_4_^+^ excess in the cytoplasm when K^+^ is limited; conversely, K^+^ application can alleviate cellular NH_4_^+^ toxicity (Hoopen et al., 2010). Supplying maize plants with Si and/or Zn significantly enhanced the chemical composition (N, P, K, Mn, and Fe) of maize under drought conditions (Abdelgalil et al., 2022). Furthermore, a significant decrease in total sulfate in roots in response to K deficiency may be explained by downregulation of important key components of the sulfate assimilation pathway in response to K deficiency (e.g., *SULTR1;1*, *APK4*, and *SOT7*), demonstrating the specificity of the K and S cross‐talk, whereas the K‐deficiency response contributed to a significant increase in the content of reduced S (Forieri et al., 2017). Furthermore, S supplementation increased Se accumulation while decreasing Fe accumulation in almost all wheat genotypes. Furthermore, Se treatment increased Fe content in wheat genotypes, implying a synergistic effect of selenate treatment on Fe accumulation (Coppa et al., 2023). Medici et al. (2019) revealed that N signaling modulate the P‐deficiency response (PSR) in rice via three molecular integrators (PHR1, PHO2, and NRT1.1). N signaling regulates N–P cross‐talk in rice by controlling the accumulation and turnover of PHR1. The phosphate2 (PHO2), an E2 ubiquitin conjugase, is an important integrator of N signaling in PSR, wherein PHO2 regulates the activities of the rice nitrate transceptor, NRT1.1, indicating that NRT1.1 is a component of N–P‐signaling cross‐talk in plants. The mechanism underlying PSR activities via N signaling is conserved in rice and wheat species (Medici et al., 2019). A more recent study found that a moderately high Zn supply increased the root‐to‐shoot translocation of N into the leaves and brown rice and increased rice yield; simultaneously, N application substantially enhanced the root‐to‐shoot translocation of Zn into the leaves and brown rice (Ji et al., 2022). Another study revealed that a high Zn concentration reduced rice yield and caused P starvation by preventing the distribution of P into leaves and the movement of P from the roots to the shoots by downregulating the Pi transporter genes *OsPT2* and *OsPT8* in shoots of rice plant (Ding et al., 2021). Contrarily, P supply reduced Zn levels in rice plants by suppressing the expression of the *OsZIPs* Zn transport gene family (Ding et al., 2021). Dai et al. (2016) reported that *OsWRKY74*, a WRKY transcription factor in rice, regulates phosphate‐deficiency tolerance and is involved in Fe‐deficiency responses. Under P deficiency, the expression of *OsWRKY74* increased in rice, resulting in higher Fe accumulation compared to the wild type. This finding suggests that *OsWRKY74* is involved in the cross‐talk between Pi‐ and Fe‐deficiency signaling. These molecular mechanisms underlying mineral nutrients cross‐talk‐mediated improved plant tolerance to changing environments are critical not only to optimize plant growth and grain yield enhancement under stress‐prone environments.

## 3 GENETIC STRATEGIES FOR INCREASING THE UPTAKE OF MINERALS UNDER STRESS‐PRONE ENVIRONMENTS AND MINERAL BIOFORTIFICATION TO ELEVATE GRAIN NUTRITIONAL CONTENT

### 3.1 Genetic regions influencing mineral uptake under stress‐prone environments

QTL mapping has emerged as an important tool for dissecting various QTLs associated with different physiological traits of the plants such as nutrient uptake, acquisition, transportation, or sequestration (Guo et al., 2012; Kong et al., 2013). A study based on QTL analysis in wheat at seedling and adult stages contained 131 recombinant inbred lines (RILs) along with two parent varieties for analyzing 20 traits ranging from physiological (K content and KUE) to agronomic parameters such as root shoot dry weight, grain number per spike resulted in identification of 87, 51, and 29 additive QTLs in hydroponic culture, pot experiment, and field experiment, respectively (Kong et al., 2013). Apparently 80%–90% of them were seen only once on different K treatments showing that these QTLs depict big variation in growth optimization parameters. The remaining additive QTLs that appear more than once can be further be used for MAS for K efficiency.

Several QTLs have been identified for P use efficiency (PUE) and other related traits in maize (Zhu et al., 2005), soybean (Li et al., 2005), common bean (*Phaseolus vulgaris*; Liao et al., 2004), and rice (Sandhu et al., 2019) during low P stress conditions. Yan et al. (2004) detected 19 QTLs under P deficiency associated with P‐uptake, root hairs, and acid exudation on eight linkage groups in two contrasting genotypes of common bean that differ in their P efficiency that could further be used in marker‐assisted breeding (MAB) for enhancing P efficiency in the crop. Ample evidence on the identification of QTLs associated with nutrient use efficiency traits under nutrient stress in plants using several traditional molecular markers such as RAPD (randomly amplified polymorphic DNA), RFLP (restriction fragment length polymorphism), AFLP (amplified fragment length polymorphism) in rice under low N (Tong et al., 2011), low P (Luo et al., 2017; Mahender et al., 2018), and K‐deficient conditions (Luo et al., 2017) has been illustrated. However, the uses of these markers require high costs and are labor‐intensive. Thus, among various molecular markers, SNP (single nucleotide polymorphism) emerges as the most efficient and reliable marker system for QTL analysis. For instance, SNP marker‐based identification of 261 putative QTLs associated with nutrient efficiency trait via selective introgression lines derived from Weed Tolerant Rice 1 (recipient) and Hao‐an‐nong (donor parent), out of which 49 major QTLs showed high phenotypic variability. Among these 49 QTLs, 22 QTLs were detected as the novel ones associated with traits of partial factor productivity and agronomic efficiency under varying rates of P and N. The fine‐tuning of hotspot QTLs suggested their expression in all the six nutrient conditions (–N, –P, –NP, –NPK, 75N, and NPK) (Jewel et al., 2019).

A total of 55 QTLs, associated with agro‐morphological and P‐content traits, were detected in finger millet under low P condition (Maharajan et al., 2023). Similarly, Gao et al. (2020) identified several QTL for PUE and P acquisition efficiency and yield‐related traits at the maturity stages in 128 RILs of barley under low P conditions (Gao et al., 2020). P efficiency was evaluated using photosynthesis traits in 219 varying soybean varieties using GWAS study and as a result, 14 novel genomic regions harboring 30 significant SNPs associated with photosynthesis‐related traits in different P concentrations were detected (Yang et al., 2020). Two major loci, that is, qP13 and qP19 were associated with both photosynthesis and low P tolerance in soybean. The resulting MTAs and associated genes can assist in further MAB for nutrition enhancement in wheat varieties. Recently, more than 400 QTLs associated with 70 traits (including micronutrient contents, agronomic traits, and grain quality) were identified in wheat (Shariatipour et al., 2021). Li et al. (2022) identified a set of 411 GWAS‐associated genes in 5 QTLs and 2722 differentially expressed genes in responsive to low N, among which *OsNIGT1*, *LOC_Os05g45020*, and *LOC_Os06g43090* were three novel strong candidates identified for N‐deficiency tolerance or NUE in rice. These findings can help in understanding the genetics of important mineral nutrients in plants and aid in genetic marker‐based identification of high nutrient genotype. Functional validation of identified genes can also aid in the development of nutrient‐rich crops without affecting plant yield. Once the associated QTLs are determined, the identification of the associated genes involved in maintaining plant nutrition followed by their pyramiding and MAB can be done to produce the desired climate resilient variety with both high yield and enhanced nutrient density.

### 3.2 Genetic regions influencing mineral enrichment in grains

The biofortification of pearl millets revealed a positive correlation between micronutrient content of Fe and Zn within the grain without any effect on seed size and grain yield (Govindaraj et al., 2013; Gupta et al., 2009). In a recent study, 23 QTLs were detected in doubled haploid lines of rice derived from IR05F102 × IR69428 rice population for 8 agronomic traits (such as yield, plant height and number of tillers), Zn and Fe concentrations using SNP markers and BLUEs (Calayugan et al., 2020). IR69428 parent donor contributed to most of the QTLs associated with enhanced Zn and Fe traits in rice, and the detected QTLs (*qZn5.1* with *OsZIP6* gene) were associated with the genes involved in metal homeostasis. Nine QTLs were stable as confirmed by QTL–environment interaction analysis out of which two were associated with Zn localized on chromosomes 5 and 12 (Calayugan et al., 2020). High Zn lines detected could further be used in the biofortification of rice for enhanced Zn content that can improve the nutritional demand of the population. Another set of experiments were conducted on a doubled haploid population of intra‐japonica cross between “Hwaseonchal” and “Goami 2” varieties of rice to identify major QTLs and related genes associated with Fe and Zn concentration (Jeong et al., 2020). Out of 21 major detected QTLs, *qFe7* and *qZn7* majorly contributed to variance in Fe and Zn content in brown rice. Co‐localized QTLs for Zn and Fe concentration identified at chromosome 1, 4, 7, and 11. The chromosome 7, an additive effect of a region near qFe7 and the effect of epistatic interaction of regions on chromosome 2 and 10 contributes to the possible correlation of Fe and Zn content. Eleven high priority genes for Zn and Fe content related to major QTLs *qFe7* and *qZn7* were also identified, which were associated with Fe and Zn homeostasis in the doubled haploid population (Jeong et al., 2020). The aforementioned outcomes suggested that the grain Fe and Zn content are regulated by genes collated with their associated major QTLs, and these QTLs can be potentially exploited for MAB for the production of high Zn and Fe grain varieties. Likewise, genetic analysis of quantitative traits reported that chromosome 6B from durum wheat is associated with high grain Zn concentration and grain yield (Cantrell & Joppa, 1991; Chee et al., 2001). Further, the genetic mapping of recombinant substitution chromosome lines produced via wild emmer as a progenitor substituting individual chromosomes into the genetic background of durum wheat cv. Langdon (LDN) (Joppa & Cantrell, 1990) revealed that all the substituted lines had high grain protein and Zn concentration (Cakmak et al., 2004). Genetic mapping identified a single locus associated with variation in grain protein concentration in durum wheat, namely, Gpc‐B1 (Olmos et al., 2003) and its multiple effects on mineral nutrient accumulation and plant phenotype (Distelfeld et al., 2007). Substituted chromosome lines with Gpc‐B1 allele show a high accumulation of Zn (12%), Mn (29%), Fe (18%) confirming the involvement of Gpc‐B1 in the remobilization of Zn, Fe, and Mn from leaves up to grains adding their role in early senescence.

Qin et al. (2012) identified several mineral QTLs that co‐localized with each other for two sets of F2:3 populations, including the QTL for Zn kernel (ZnK), Zn concentration (ZnC), Fe kernel (FeK), and Fe concentration (FeC) on chromosome 2, QTL for Znk, FeK, and FeC on chromosome 9, and QTL for ZnK and ZnC on chromosome 7. Furthermore, Lung'aho et al. (2011) identified three modest QTL for grain Fe concentration (FeGC) and 10 QTLs for grain Fe bioavailability (FeGB) from an Intermated B73 × Mo17 (IBM) recombinant inbred population of maize. Unlike Fe and Zn, QTL mapping studies for grain selenium (Se) concentration in crop plants are uncommon. Yan et al. (2018) used an RIL population derived from a cross between *Triticum dicoccoides* (accession G18‐16) and Langdon (Durum wheat) to map a total of 15 QTLs on chromosomes 1A, 1B, 2B, 3A, 4B, 5A, 6A, 7A, and 7B, explaining 1.4%–18.6% of the phenotypic variation for GSeC (grain Se concentration) and GSeY (grain Se yield). Wang et al. (2017) used an RIL population derived from a cross between two Chinese winter wheat varieties (Tainong18 and Linmai6) to map 16 QTLs (seven at the seedling stage and nine at the adult stage) for 6 Se content‐related traits on 8 chromosomes, namely, 1B, 2B, 4B, 5A, 5B, 5D, 6A, and 7D, under both field‐grown and hydroponic conditions. These findings identified a number of main‐effect QTLs and their associated markers that can be used in the MAS program for Se biofortification of wheat grain.

GWAS has been emerging as an asset to pace up the process of enhancement of nutritional density in plants by overcoming the limitations of traditional QTL mapping.

Through GWAS and targeted association studies, we found association peak on chromosome 7 within 59.61 kb with candidate genes OsLonP3, OsDLN197, and LOC_Os07g49020 exhibiting significant PVEs for grain Fe and Zn accumulation in brown rice. In addition, donor lines containing haplotypes (CCTGT) derived from key candidate genes OsNAS3 and OsZIP5 exhibit 10.57 ppm mean Fe and 20.32 mean Zn (Pasion et al., 2023). This study identified 109 high‐value target genes associated with multiple minerals in brown rice using high density 1.09 million SNPs (Pasion et al., 2023). Biofortification efforts of wheat germplasm in CIMMYT with targeted breeding to improve Zn and Fe levels have also been very promising (Velu et al., 2018). Characterization of Zn concentration trait in 330 bread wheat lines using GWAS revealed that 39 MTAs (marker trait associations) along with 2 major SNPs on chromosome groups 2 and 7 found to associate with the trait of grain Zn concentration (Velu et al., 2018). The QTLs for high grain Zn and Fe trait in wheat identified on chromosomes 2 and 7 with genes for nutrient uptake and translocation (Crespo‐Herrera et al., 2017; Hao et al., 2014). Novel candidate regions along with the genes of metal homeostasis maintenance were also identified on chromosomes 2B and 7B, and it was indicated that zinc motifs and phosphatase play an additive role in Zn accumulation within wheat grain (Velu et al., 2018).

GWAS study for genetic dissection of five nutrients, that is, Zn, Fe, Cu, Mn, and P, using three different multi‐locus mixed GWAS models for immature grains, mature grains, and immature rachis of wheat were performed, where candidate genomic regions were detected that significantly harbors co‐localized MTAs (279 in mature grain, 379 in immature grain, and 481 in immature rachis), on chromosomes 1A, 3B, and 5B, including key genes involved in nutrient uptake, their transport, and accumulation (Cu et al., 2020). Furthermore, GWAS study identifies Zn–NA (nicotianamine) transporter gene *ZmYSL2* that is responsible for loading Zn to maize kernels. Overexpression of *ZmYSL2* increases the Zn concentration in the kernels, a validated candidate gene for Zn biofortification of maize (Chao et al., 2023). In addition, nine putative QTLs for Fe, Zn, and protein content in grains and thousand kernel weight were identified, covering candidate genes such as P‐loop containing nucleoside triphosphate hydrolase, nodulin‐like protein, NAC domain, purine permease, zinc‐binding ribosomal protein, cytochrome P450, protein phosphatase 2A, zinc finger CCCH‐type, and kinesin motor domain located within the identified QTL regions responsible for the regulation of Fe homeostasis, Zn transportation, Fe, Zn, and protein remobilization to the developing grain and increased NUE in wheat (Jadon et al., 2023).

## 4 DEPLOYING BREEDING AND BIOTECHNOLOGICAL APPROACHES TO ADDRESS NUTRITIONAL SECURITY UNDER AMBIENT AND CHANGING CLIMATES

**FIGURE 3:**
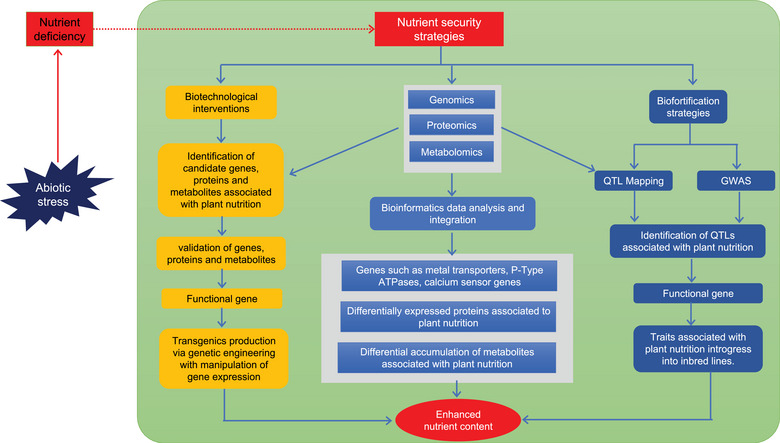
Strategies for crop improvement to enhance nutritional levels for sustainable agriculture. Several strategies have emerged for crop improvement to enhance nutritional levels in plants to cope with nutritional deficiency during abiotic stress conditions for sustainable agriculture. Biotechnological interventions backed up by biofortification of crops and other agronomic strategies have been employed for producing nutritionally rich crops with enhanced tolerance to abiotic stresses. Biotechnological approaches involve identification and manipulation of genes, proteins and metabolites associated with plant nutrition, whereas approaches such as quantitative trait locus (QTL) mapping and genome wide association studies (GWAS) have also been employed for biofortification of food crops. Other agronomic traits include the utilization of several nutrient rich fertilizers, manures, foliar application of nutrients and/or different system of agriculture such as intercropping to prevent nutrient deficiency.

To overcome malnutrition concern among the global population, it is necessary to produce crops that are dense in nutrients to feed the rapidly increasing population overcoming the barrier of nutrient deficiency. During the past more than a decade, serious efforts have been made for breeding crop varieties that are biofortified for enriching Fe and Zn mineral nutrients. These efforts involved both the conventional breeding and, in limited cases, deploying the MAB approaches. Other novel approaches (including transgenics and genome‐editing) have been continuously emerging as efficient tools for improving the nutrient status of the plant to ameliorate sustainable crop production (Table 2; Figure 3). Climate change has severe impact on minimizing yield losses as well as impacting nutrient uptake by the plants (FAO, 2017). In this context, we need to identify strategies to develop biofortified varieties amenable to grow in different ecologies under changing climatic conditions.

**TABLE 2:** Strategies for nutrient enhancement via biotechnological, biofortification, and agronomic approaches.

**Biotechnological interventions**				
**Plant species**	**Gene manipulated**	**Effect on nutritional traits**	**Other plant traits affected**	**Reference**
*Solanum lycopersicum* cv. “moneymaker” (tomato)	*codA*	Enhanced Pi uptake, translocation capacity and maintain Pi homeostasis	High shoot sucrose levels, increased photosynthesis, root development, and enhanced crop yield	Li et al. (2019)
*Oryza sativa* (rice)	*OsNRT2.3b*	Enhanced N, Fe, and P uptake	Improved nitrogen use efficiency and grain yield	Luo et al. (2020)
*Triticum aestivum* (wheat)	*EdVP1*	Enhanced K uptake from the roots	Increased plant height, biomass, high grain number per spikelet, tiller number per plant, improved grain yield	Zhou et al. (2020)
*Nicotiana tabacum* (tobacco)	*PmPT1*	Increased P accumulation in the roots and shoots	Increased dry weight, enhanced chlorophyll, soluble sugars, soluble proteins content and increased activity of POD, SOD and CAT, and decreased MDA levels	Zhang, Hong et al. (2020)
Tobacco	*HvAlaAT*	Increased N levels	Enhanced levels of total amino acids, crude proteins, stalk diameter, leaf area, and seed weight	Ahmed et al. (2020)
Wheat	*TaDof1*	Modulation in C and N metabolism, improved N utilization	Increased number of spikes, spike length, grain number and grain weight, increased chlorophyll content, soluble protein and soluble sugar, enhanced growth	Hasnain et al. (2020)
*Arabidopsis thaliana* (Arabidopsis) and rice	*SiMYB3*	Enhanced N uptake	Enhanced root development by regulating auxin synthesis resulting in plant growth	Ge et al. (2019)
**Biofortification**				
**Plant species**	**Associated markers/traits/locii**	**Effect on nutritional traits**	**Other associated traits**	**Reference**
Rice	*qZn_1.1_ + qZn_5.1_ + qZn_9.1_ + qZn_12.1_* *qFe_9.1_*, *qFe_12.1_*	Zn level in grains Fe level in grains	Increased grain yield	Calayugan et al. (2020)
Rice	*Fe3.3* and *qFe7.3* *qZn2.2*, *qZn8.3* and *qZn12.3*	Increased Fe content Increased Zn content	Higher grain yield	Pradhan et al. (2020)
Rice	*qFe7* and *qZn7*	Increase in Fe and Zn content	Higher grain yield	Jeong et al. (2020)
Rice	Eight QTLs on chromosomes 2, 4, 5, and 6	N, P, Fe uptake	Enhanced root architecture and grain yield	Sandhu et al. (2019)
RILs derived from *Triticum boeoticum× Triticum monococcum*	*QFe.pau‐2A* and *QFe.pau‐7A* *QZn.pau‐7A*	Accumulation of Fe in grain	No study for correlation with other traits	Tiwari et al. (2009)
RILs bred from the cross *Triticum spelta* × *T. aestivum*	*QZn.bhu‐2A* *QZn.bhu‐2B* *QZn.bhu‐3D* *QZn.bhu‐6A* *Zn.bhu‐6B* *QFe.bhu‐1A.1* *QFe.bhu‐1A.2* *QFe.bhu‐1A.3* *QFe.bhu‐2A* *QFe.bhu‐3B*	Accumulation of Zn in grain Accumulation of Fe in grain	Improvement of grain nutrient content and accumulation of Zn and Fe show positive correlation (rp = 0.79**)	Srinivasa et al. (2014)
RILs derived from the cross *Triticum dicoccoides* (accession G18‐16) and durum wheat cultivar Langdon (LDN)	15 QTLs on chromosomes 1A, 1B, 2B, 3A, 4B, 5A, 6A, 7A, and 7B	Accumulation of Se in grain	Higher grain yield	Yan et al. (2018)
RILs derived from the cross between two Chinese winter wheat varieties (Tainong18 and Linmai6)	16 QTLs on chromosomes 1B, 2B, 4B, 5A, 5B, 5D, 6A, and 7D	Accumulation of Se in seedling and grain	Enhanced Se uptake and accumulation	Wang et al. (2017)
Intermated B73 × Mo17 (IBM) RI population of maize	3 QTLs for FeGC and 10 QTLs for FeGB on chromosome 3, 6, and 9	Grain Fe concentration (FeGC) and grain Fe bioavailability (FeGB)	Higher yield	Lung'aho et al. (2011)
F_2:3_ populations derived from the cross Mu6 × SDM (MuS) and Mo17 × SDM (MoS) of maize	Several QTLs for Zn kernel (ZnK), Zn concentration (ZnC), Fe kernel (FeK) and Fe concentration (FeC) on chromosome 2, QTL for ZnK, FeK and FeC on chromosome 9 and QTL for ZnK and ZnC on chromosome	Increasing Zn and Fe concentration in maize kernel	Higher yield	Qin et al. (2012)
**Agronomic strategies**				
**Plant species**	**Approaches employed**	**Effect on nutritional traits**	**Plant traits affected**	**Reference**
Maize	Biofertilizer mixture (*Azotobacter chrocoocum*, AMF, and *Bacillus circulans*)	Enhanced levels of N, P, and K in leaves and roots	Increased the grain quality and metabolism, improved nutritional status increased plant height, root and shoot dry weight, chlorophyll content, and leaf area	Gao et al. (2020)
Wheat	K application along with foliar‐application of micronutrients such as Zn, Fe	Increased accumulation of K, Fe and Zn in the grain	Increased grain yield with high nutritive value	Hussain et al. (2020)
Maize	PGPR strain *Kocuria rhizophila* Y1	Enhanced contents of P, N, Ca^2+^ and K^+^, high K^+^/Na^+^	Enhanced growth parameters such as root and shoot length, root and shoot dry weight, improved photosynthetic performance, IAA content, antioxidative capacity	Li et al. (2020)
Maize	Application of N fertilizer under PRI	Increased N uptake	Enhanced grain yield, harvest index, and root length density	Qi et al. (2020)
*Vigna radiata* (mungbean)	Zn osmopriming + foliar application	Enhanced grain Zn concentration	Amelioration in root length, leaf area per plant, crop growth rate, and net assimilation rate, increased grain yield	Haider et al. (2020)
Maize	ZnSO_4_ treatments via soil application, foliar application	Enhanced Zn content	Increased grain yield	Tariq et al. (2014)
*Sorghum bicolor* (sorghum)	Biofertilizers *Azospirillum* and PSB	Enhanced P and N levels	Improved grain yield and protein content	Patidar and Mali (2004)

Abbreviations: AMF, arbuscular mycorrhizal fungi; C, carbon; Ca, calcium; CAT, catalase; Fe, iron; IAA, indoleacetic acid; K, potassium; MDA, malondialdehyde; N, nitrogen; P, phosphorus; POD, peroxidase; PRI, partial root‐zone irrigation; PSB, phosphate‐solubilizing bacteria; QTL, quantitative trait locus; RI, recombinant inbred; RILs, recombinant inbred lines; SOD, superoxide dismutase; Zn, zinc; ZnSO_4_, zinc sulfate. **P ≤ 0.01.

### 4.1 Conventional breeding

By identifying suitable donor genetic resources such as synthetic, wild, and landraces exhibiting higher Fe and Zn content with enhanced bioavailability, the conventional breeding approaches have been taken up to bred biofortified crops. Diploid progenitors of hexaploid wheat (*Aegilops tauschii*), wild emmer (*T. dicoccoides*), einkorn (*Triticum monococcum*), *Triticum spelta*, *Triticum polonicum*, and *T. aestivum* landraces are the most promising high Zn and Fe sources. Among the wild wheat collections tested so far, *Triticum turgidum* ssp. dicoccoides showed a significant genetic variation in Zn ranging from 14 to 190 mg kg^−1^ and Fe up to 88 mg kg^−1^ (Cakmak et al., 2010). Translocation from various *Aegilops* spp. and rye (*Secale cereale*) to the Pavon 76 background produced several synthetic hexaploids (SHW), *T. spelta*, and several pre‐breeding lines with greater variation in Zn (38–72 mg kg^−1^) and Fe content (32–52 mg kg^−1^) (Velu et al., 2018). The SHWs and their derivatives were particularly useful, for transfer of genes for high Zn and Fe, from diploid and tetraploid progenitors of wheat to produce high yielding biofortified wheat lines. In addition to the above, the team at CIMMYT also worked in collaboration with National Agriculture Research Systems (NARS) for releasing eight varieties in India: WB 02, HPBW 01, Pusa Tejas (HI 8759), Pusa Ujala (HI 1605), MACS 4028 (durum wheat). Similarly, a Zn‐biofortified variety “Zincol2016” was released in Pakistan. These varieties have up to 42 mg kg^−1^ Zn and up to 46.1 mg kg^−1^ Fe, which is 20%–40% higher than the level of Zn in local varieties (Saini et al., 2020; Singh & Velu, 2017). Overall, there has been a significant reward of using SHWs for the development of biofortified wheat varieties, suggesting their continued importance in breeding for biofortification. Rice varieties with increase in endosperm Zn content (18–20 mg kg^−1^) have been developed (Swamy et al., 2016) by leveraging natural rice genetic variation in Zn concentrations ranging from 15.9 to 58.4 mg kg^−1^ in unpolished grains (brown rice) (Graham et al., 1999). However, the natural genetic variation for grain Fe concentrations is limited (7.5–24.4 mg kg^−1^) in unpolished brown rice (Graham et al., 1999). Moreover, as a large portion of the grain Fe in brown rice is found in the aleurone layer that is removed by milling (Johnson et al., 2011), the Fe concentration in the polished grain (endosperm) is at least 50% less (Vasconcelos et al., 2003). Considering the narrow genetic variation for Fe concentrations in rice grains, conventional breeding is difficult to achieve the required target Fe concentration in polished grains. Furthermore, the negative correlation between yield and nutrient traits makes it even more difficult to develop nutritious varieties with stable yields (Anandan et al., 2011).

### 4.2 Marker‐assisted breeding

The transfer of the high grain protein content gene *Gpc‐B1* is one of the notable examples of successful cases, where MAB was used to improve Zn and Fe. Near‐isogenic lines of NAM‐B1 showed a modest increase (18%) in Fe content because this gene is closely linked to loci for high Zn and Fe. In more than a dozen studies the gene *Gpc‐B1* has been introgressed into both tetraploid and hexaploid wheats (Gupta et al., 2020). This led to the development of numerous hexaploid wheat and durum wheat cultivars in the USA, Canada, and other countries. Zn content was significantly higher in 93% of the high GPC lines (an increase of 11.6 mg kg^−1^ on average; Tabbit et al., 2017). Additionally, there are markers that can be used in MAB to decrease phytic acid (PA) and increase bioavailability in maize (Yathish et al., 2023). Additionally, molecular markers were used to assess the genetic diversity of rice grain Fe and Zn levels in the representative groups of native and exotic rice accessions. When compared to coarse grain accessions, aromatic rice accessions had higher levels of Fe and Zn (Raza et al., 2020). Thus, MAB is helpful in increasing the effectiveness of selection early in the breeding cycle and aiding in the improvement of target traits.

### 4.3 Transgenic approaches

**FIGURE 4:**
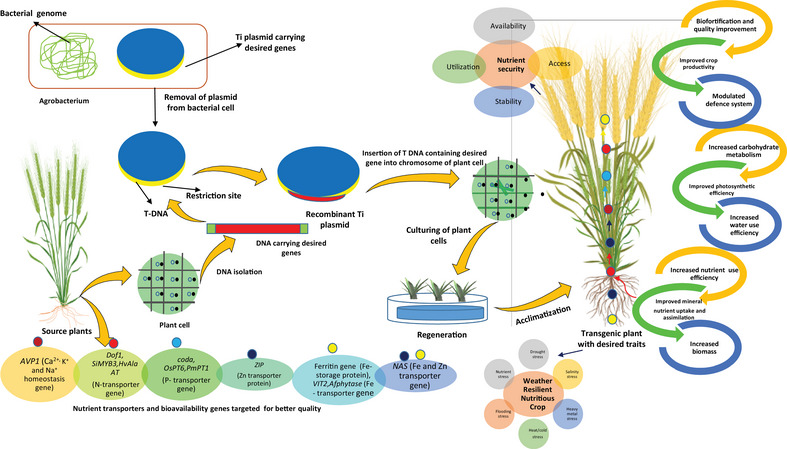
Generation of engineered plants by modulating the expression of genes associated with nutrient uptake, their transportation and distribution can aid in maintaining nutritional homeostasis and improve plant growth under abiotic stressed conditions. Several stress‐responsive genes, including mineral transporters and bioavailability genes *AVP1* (K^+^ and Na^+^ homeostasis gene); *Dof1*, *SiMYB3*, *HvAlaAT* (N‐transporter gene); *coda*, *OsPT6*, *PmPT1* (P‐transporter gene); *ZIP* (Zn‐transporter protein); Ferritin (Fe‐storage protein), *VIT2*, *Afphytase* (Fe‐transporter gene); *NAS* (Fe‐ and Zn‐ transporter gene) regulate nutrient uptake, acquisition, and their metabolism in plants have been identified and manipulated by genetic engineering approaches through agrobacterium‐mediated genetic transformation to produce transgenics with improved nutritive values and abiotic stress tolerance.

Engineering metabolic pathways by reinforcement of gene identification associated with nutritional quality traits and modifying their expression using appropriate molecular approaches have emerged as an important factor for ameliorating malnutrition. Generation of engineered plants by modulating the expression of genes associated with nutrient uptake, their transportation and distribution can aid in maintaining nutritional homeostasis and improve plant growth under normal and stressed conditions (Figure 4). For instance, overexpression of *AVP1*, an H^+^‐pyrophosphatase gene (from Arabidopsis) enhances the accumulation of sodium (Na^+^) and Ca^2+^ within the leaves and roots of transgenic alfalfa (*Medicago sativa*), although K^+^ levels reduce significantly during salinity treatment, and the decrease in transgenic lines has been lower than that in WT (wild type; Bao et al., 2009). However, under drought stress conditions, the overexpression of *AVP1* in alfalfa resulted in significant accumulation of all the three cations (Na^+^, K^+^, and Ca^2+^) in transgenic lines. This study deciphered the role of *AVP1* in maintaining intracellular K^+^ and Na^+^ homeostasis, which has a critical involvement in plant adaptation against drought and salinity stress (Niu et al., 1995). Under N‐deficit conditions, overexpression of maize *Dof1*, an activator of genes associated with the metabolism of organic acids enhances plant growth and amino acid levels (glutamine and glutamate), reduces glucose levels, improves N‐assimilation, and elevates N‐content in Arabidopsis (Yanagisawa et al., 2004). *ZmDof1* overexpression elevates N and C levels with improved photosynthetic rate and biomass in transgenic rice (Kurai et al., 2011). Additionally, co‐overexpression of the N transport, assimilation, and utilization genes (*OsAMT1;2*, *OsNPF8.9a, OsNR2*, and *OsAS1*) increases rice grain yield and NUE (Luo et al., 2023). In connection with this, it was revealed that the overexpression of *OsAMT1;2* promoted an increase in rice biomass and NH_4_^+^ uptake under NH_4_^+^‐limited conditions (Konishi et al., 2021). The overexpression of *STP13* (a hexose transport protein‐mediated by NO_3_^−^‐induced GATA TF) increases glucose uptake with simultaneous enhancement in N‐content and increased biomass in Arabidopsis under N deficiency (Schofield et al., 2009). Furthermore, the overexpression of *O. sativa* plasma membrane (PM) H^+^‐ATPase 1 (*OSA1*) promoted NH_4_^+^ uptake and assimilation in roots and enhanced light‐induced stomatal opening with higher photosynthesis rate leading to higher NUE and grain yield (Zhang, Wang, et al., 2021). Similarly, core autophagy gene in rice, *OsATG8b* confers tolerance to N starvation and increases yield and NUE in Arabidopsis (Zhen et al., 2019).

P is a macronutrient that is indispensable for normal plant growth and forms an integral part of adenosine triphosphate (ATP), inorganic phosphate (Pi), adenosine diphosphate (ADP), and nicotinamide adenine dinucleotide phosphate hydrogen that provide energy to plant cells for various metabolic and cellular processes and thus its deficiency can also limit the crop yield. P acquisition and its accumulation can be improved by overexpressing phosphate transporter genes in transgenic plants as observed in rice (Park et al., 2007) and wheat (Guo et al., 2013) that can positively regulate plant growth. Yan et al. (2014) demonstrated that a P‐transporter gene from rice, that is, *OsPT6* improved P efficiency in soybean during low P stress conditions via genetic engineering. The overexpression of rice *OsPT6* gene in soybean increased P accumulation together with the significant increase in plant growth‐related parameters, including plant height, root weight and length, number and size of seeds that ultimately ameliorated plant growth during P‐deficit conditions. The overexpression of *codA* gene in tomato significantly enhanced Pi uptake, translocation capacity, and aided in the maintenance of Pi homeostasis and resulted in high shoot sucrose levels, increased photosynthesis, root development, and enhanced crop yield (Li et al., 2019). Similarly, overexpressing *PmPT1*, a phosphate transporter increases P accumulation in the roots and shoots, dry weight, enhances chlorophyll levels, soluble sugar and protein contents, and activity of antioxidant enzymes along with decreased MDA levels (Zhang, Hong, et al., 2020).

Tissue‐specific enhancement of Fe content in rice grains was obtained up to threefolds in transgenic rice by the introduction of ferritin gene from soybean under the regulation of GluB‐1, a rice seed‐storage protein glutelin promoter (Goto et al., 1999). Similarly, Fe content in transformed tobacco leaves was also increased up to 1.3‐folds by the expression of ferritin gene from soybean under the control of CaMV 35S promoter (Goto et al., 1998). This data suggests that the overexpression of ferritin genes in plants such as rice and tobacco can lead to appreciable enhancement in Fe content by regulating the expression of Fe storage proteins such as ferritin. Interestingly, ferritin regulation has been found to be involved in mediating acclimation responses in plants under salt stress and oxidative stress (Xi et al., 2011; Zang et al., 2017). In Zn‐ and Fe‐deficient conditions, the expression of the *NAS* (*nicotinamide synthase*) gene significantly induced tolerance against Fe and Zn deficiency in rice grains (Lee et al., 2009). Activation of *OsNAS3* in the transgenic lines led to the increased Fe and Zn levels, elevated chlorophyll content, enhanced plant growth, and improved tolerance against nickel (Ni), Zn and Cu. Conversely, *osnas3‐1*, the knockout mutants of the *NAS* gene significantly reduced Fe, Zn, and Cu levels in rice seeds suggesting the role of the *NAS* gene in maintaining mineral nutrient homeostasis (Lee et al., 2009). More importantly, the targeted expression of three transgenes (*AtNAS1*, *Afphytase*, and *Pvferritin*) in rice grains under the control of endosperm specific promoter increased the Fe (by 4.5–5.3‐folds) in Fe‐deficient conditions and altered Zn homeostasis by its accumulation (1.3–1.5‐fold higher) in the endosperm of the transgenic rice (Wirth et al., 2009). Enhancement of Fe content was also achieved in wheat and barley by overexpressing *TaVIT2* in wheat and barley endosperm under the control of endosperm specific promoter (Connorton et al., 2017). The overexpression of *TaVIT2* caused an increase in Fe content (by twofolds) in the wheat endosperm, whereas in barley both Fe and Mn contents were increased in whole grain. Plant *VITs* have also been found to be involved in regulating Fe homeostasis, N metabolism, and NH_4_^+^ assimilation (Cao, 2019), indicating the importance of the gene family as a promising candidate for nutrients management in plants. In addition, the modulation of nutrient transporters that facilitate nutrient uptake and transportation can effectively enhance nutrient content within the plant. For instance, Zn transporter proteins such as ZIP may contribute to maintaining Zn homeostasis by regulating its uptake and intracellular transportation (Guerinot, 2000) as studied in rice (Ishimaru et al., 2005) and Arabidopsis (Plaza et al., 2007). Plants with high Zn content can be engineered by the expression of ZIP transporters under the control of specific promoters for a genetic enhancement of the nutrient content in deficient plants. Recently, a significant increase in Zn content in finger millet (*Eleusine coracana*) was obtained by overexpressing *OsZIP1* under the control of 35S constitutive promoter and Bx17, an endosperm‐specific promoter (Ramegowda et al., 2013). Thus, the overexpression of *NAS* genes makes nicotianamine an interesting target for Zn biofortification. Thus, modifying the nutritional status of the plants using transgenic approaches has facilitated plant growth, increased nutritional content in the grains for addressing malnutrition, and increased productivity to assist in sustainable agriculture programs.

Phytate is a known anti‐nutrient compound that mostly accumulates in the aleurone layer. It has six negatively charged ions and can chelate divalent cations such as Ca^2+^, Fe^2+^, Mg^2+^, and Zn^2+^, eventually leading to low bioavailability of these minerals. This can be addressed by manipulating the PA biosynthetic pathway through RNA interference (RNAi)‐mediated silencing of key enzymes. The three targeted enzymes were myo‐inositol‐3‐phosphate synthase, inositol‐1,3,4,5,6‐pentakisphosphate 2‐kinase (IPK1), and inositol triphosphate kinases (ITPK) homolog (OsITP/6K‐1), resulting in a 1.3–1.8‐fold increase of Fe in milled rice (Ali et al., 2013b, 2013a; Karmakar et al., 2020). In addition, RNAi‐mediated downregulation of *ITPK‐2* has resulted in enhanced inorganic P along with Zn^2+^ and Fe^2+^ in transgenic rice (Sengupta et al., 2021). Increased Fe translocation in rice endosperm was also achieved by the overexpression of Fe transporter gene *OsYSL2* (Masuda et al., 2013). Overexpression of *OsYSL2* under the control of the *OsSUT1* (sucrose transporter 1) promoter sequence showed a significant increase in the accumulation levels of Fe, Zn, Mn, and Cu in the seeds of field‐grown rice (Masuda et al., 2013). Similarly, the overexpression of *Arabidopsis AtIRT1*, *AtNAS1* (nicotinamine synthase 1), and bean ferritin in rice caused higher Fe and Zn accumulation (Boonyaves et al., 2017). Alternatively, the phytase enzyme (*Afphytase*) from *Aspergillus fumigatus* can catalyze the hydrolysis of phytate and eventually release the chelated minerals (Boonyaves et al., 2016; Wirth et al., 2009). Overexpression of the *appA* (phytase) gene from *Escherichia coli* resulted in two‐ and threefold increases in Fe and Zn, respectively (Bhattacharya et al., 2019). The introduction of *Afphytase* into the rice endosperm with Cys‐rich metallothionein‐like protein leads to an increase in Fe content (Lucca et al., 2001). The enhancement of iron availability may be attributed to the increase in Cys concentration in the endosperm (Majumder et al., 2019).

### 4.4 Genome editing approaches

Genome editing cutting‐edge biotechnological techniques offers a new paradigm for undertaking target‐specific precision editing to engineer a plant's genome. The recent advancement of CRISPR/Cas9‐based genome editing technologies in crops has emerged as a new generation breeding tool in the development of climate resilient crops, enhancement of nutrient content with optimized yield in crop plants (Ceasar et al., 2022; Sathee et al., 2022). Alam et al. (2022) used the CRISPR/Cas9 system to knock out the *OsbHLH024* gene in rice that resulted in increase in the levels of Ca^2+^, Zn^2+^, and Mg^2+^ in the shoot and root, as well as the expression of ion transporter genes *OsHKT1;3*, *OsHAK7*, and *OsSOS1*, thereby improving salt tolerance. Achary and Reddy (2021) used CRISPR/Cas9 to mutate the rice *GW2* gene, which improved grain architecture and nutritional (Fe, Zn, K, P, Ca) quality of the rice aleurone layer and grains. Ibrahim et al. (2022) reported increased Fe and Zn content of wheat through genome engineering of the *TaIPK1* gene, implying that *TaIPK1* plays an important role in wheat biofortification. In rice, *OsZIP9*, a member of Zn‐IRT‐related protein, has been knocked out by the CRISPR/Cas9 system. The knockout mutants had lower Zn content in roots and shoots, indicating that OsZIP9 functions as a flux carrier in rice (Huang et al., 2020). Song et al. (2022) used CRISPR/Cas9 to successfully reduce the PA content in soybean seeds, thereby improving their nutritional value.

Santosh Kumar et al. (2020) used CRISPR/Cas9 to engineer mutant alleles of the *drought and salt tolerance* (*DST*) gene in the indica mega rice cultivar MTU1010, resulting in wider leaves, lower stomatal density, improved mineral nutrient profiling, and increased grain yield under salt and drought stress. Under N‐limiting conditions, yield potential can be increased by using CRISPR/Cas9 to manipulate an *ARE1* ortholog (*the rice abnormal cytokinin response1 repressor1*) related to NUE in wheat (Zhang, Zhang, et al., 2021). Similarly, numerous N transporter genes have been genetically engineered in rice to improve its NUE, for instance, low‐affinity nitrate transporter *NRT1.1b* (Kant, 2018), high‐affinity nitrate transporter *NAR2.1*, *NRT2.3a* (Chen et al., 2020), and ammonium transporter AMT1.1 (Ranathunge et al., 2014). Furthermore, under Fe deficiency, *OsNramp5* knockout mutant lines in rice exhibited substantial enhancement in the expression of Fe/Cd transporter genes such as *OsIRT1* and *OsIRT2* (Takahashi et al., 2014). Furthermore, *osnramp5* mutant rice showed decreased Cd accumulation in seedlings as well as improved growth and developmental response (Tang et al., 2017). Furthermore, wheat genes were targeted with CRISPR/Cas9, such as *TaMADS29*‐linked with grain length and dense‐erect panicle, and plants with mutations in six alleles were generated, showing increased grain nutrient content and thousand‐kernel weight in wheat plants (Liu et al., 2023). Under low phosphorus conditions, increased spikelet number and higher grain yield were shown by knockout mutants of rice developed through in‐frame mutation into the coding region of rice *TEOSINTE BRANCHED1* (*OsTB1*), using CRISPR/Cas9 (Ishizaki et al., 2023). Over the next few years, there will likely be a sharp increase in the number of reports demonstrating the use of CRISPR/Cas9 to achieve ideotype by simultaneously targeting numerous genes in crop plants to increase the uptake of nutrients under abiotic stress treatments and target key genes to manipulate grain mineral nutrient content.

## 5 CONCLUSION AND FUTURE PROSPECTIVE

Nutrient acquisition, distribution, and availability within the plant is a critical determinant of plant growth and yield during both optimal and stress conditions. Plants are exposed to several environmental perturbations in their entire life cycle that not only deteriorate their cellular integrity but also disturb their nutritional homeostasis causing to yield loss. Stress conditions reduce nutrient uptake from the soil, their acquisition, and distribution leading to nutrient deficiency, thereby posing a greater threat to plant productivity. To overcome these limitations, we need to (i) tap the genetic wealth to identify important donors to enrich key minerals in combinations of multi‐stress treatments, (ii) deploy novel multi‐omics technologies to identify target genes to enrich minerals, minimize the yield gap, (iii) enrich superior alleles or haplotypes through haplotype enriched breeding technologies, and (iv) deploy genome edited or genetic engineering technologies to engineer rate limiting transporters to address one health strategy for maintaining plant health and productivity along with improved minerals in the grains and human nutrition.

Biofortification has several advantages over other strategies to combat micronutrient deficiencies, such as supplementation and food fortification. Biofortification is more cost‐effective, sustainable, and scalable than the supplementation of capsules or food fortification due to larger investments required for food processing. Moreover, biofortification can enhance the nutritional value of crops without changing their appearance, taste, or cooking properties, which may increase consumer acceptance and adoption. However, biofortification also faces some challenges and limitations. One challenge is to ensure that biofortified crops have better agronomic performance and higher yield stability when prone to various abiotic stresses. Another challenge is to demonstrate the efficacy and effectiveness of biofortified crops in improving human health and nutrition outcomes through rigorous trials and evaluations. To mainstream biofortified traits through genome editing and transgenic approaches important policies needs to be implemented to comply with regulatory aspects.

## AUTHOR CONTRIBUTIONS

**M. Iqbal R. Khan**: Conceptualization; writing—original draft; writing—review and editing. **Faroza Nazir**: Software; writing—original draft; writing—review and editing. **Chirag Maheshwari**: Writing—review and editing. **Priyanka Chopra**: Writing—original draft. **Himanshu Chhillar**: Writing—original draft. **Nese Sreenivasulu**: Funding acquisition; software; writing—review and editing. All authors have read and agreed to the published version of the manuscript.

## CONFLICTS OF INTEREST STATEMENT

Authors declare that they have no conflict of interest.

## Data Availability

Data availability or sharing is not applicable to this review article as no data was generated.
